# Assessing knowledge and behavioural changes on maternal and newborn health among mothers following post-earthquake health promotion in Nepal

**DOI:** 10.1371/journal.pone.0220191

**Published:** 2019-07-25

**Authors:** Rolina Dhital, Ram Chandra Silwal, Padam Simkhada, Edwin van Teijlingen, Masamine Jimba

**Affiliations:** 1 Department of Community and Global Health, Graduate School of Medicine, University of Tokyo, Hongo, Bunkyo-ku, Tokyo, Japan; 2 Green Tara Nepal, GPO, CPC, Kathmandu, Nepal; 3 Faculty of Education, Health and Community, Public Health Institute, Mount Pleasant, United Kingdom; 4 Bournemouth University, Faculty of Health & Social Sciences, Bournemouth, United Kingdom; Tribhuvan University Institute of Medicine, NEPAL

## Abstract

**Introduction:**

Disasters can disrupt the existing health system affecting the whole population, but especially vulnerable people such as pregnant women, new mothers and their babies. Despite the global progress in maternal, newborn and child health (MNCH) programmes over the years, emergency responses after a disaster are often poor. Post-disaster health promotion could play an important role in improving MNCH outcomes. However, evidence remains limited on the effect of post disaster health promotion activities in low-income countries such as Nepal.

**Methods:**

This is an uncontrolled before and after study conducted in Dhading district which was severely affected by the 2015 earthquake in Nepal. The study participants were mothers who had a child in the previous 12 months. The intervention was implemented between 2016 and 2018 and included community-engagement health promotion activities where the local stakeholders and resources were mobilized. The outcome variables included: knowledge of danger signs of pregnancy, childbirth and in newborns; and behaviours including ever attending antenatal care (ANC), a minimum of four ANC sessions and having an institutional delivery. Data were analysed using chi-squared tests, independent sample t-tests and multiple logistic regression models.

**Results:**

In total 364 mothers were recruited in the pre-intervention group and 377 in the post-intervention group. The post-intervention group was more likely to have knowledge of at least three danger signs in pregnancy (AOR [Adjusted Odds Ratio] = 2.96, P<0.001), at least three danger signs in childbirth (AOR = 3.8, P<0.001), and at least five danger signs in newborns (AOR = 1.56, P<0.001) compared to the pre-intervention group. The mothers in the post-intervention group were also more likely to ever attend ANC (AOR = 7.18, P<0.001), attend a minimum of four ANC sessions (AOR = 5.09, P<0.001), and have institutional deliveries (AOR = 2.56, P<0.001). Religious minority groups were less likely to have knowledge of all danger signs compared to the majority Hindu group. Mothers from poorer households were also less likely to attend four ANC sessions. Mothers with higher education were more likely to have knowledge of all the danger signs. Mothers whose husbands had achieved higher education were also more likely to have knowledge of danger signs and have institutional deliveries.

**Conclusion:**

Health promotion intervention helped the disaster-affected mothers in improving the knowledge and behaviours related to MNCH. However, the vulnerable population would need more support to gain benefit from such intervention.

## Introduction

Natural disasters take place often unpredictably and unexpectedly. They can destroy lives, homes, livelihoods or even whole communities, causing serious physical and psychological damage [1]. Once they occur, disasters such as earthquakes, floods or volcano eruptions can disrupt existing health systems affecting the population including pregnant women, new mothers and newborns [2]. An estimated 60% of preventable maternal deaths take place in disaster settings [2, 3]. Moreover, 15% of pregnant women experience obstetric complications and 53% of deaths among under-5 children are preventable during disasters [3].

Despite the progress in maternal, newborn and child health (MNCH) programmes over the past decade, a huge gap persists in addressing MNCH in post-disaster settings [4]. The emergency responses are often too slow [5], and especially low-income countries face challenges to address MNCH years after the disaster [6]. People are slowly beginning to realize that the health promotion activities in post-disaster settings can help improve MNCH and also encompass long-term consequences.

Health promotion is the process of facilitating people to have control over, and improve, their health [7]. Such interventions appear to be more effective when they involve the local organizations and local resources [8]. Enhancing the capacity of local resources can be self-sustaining in the long run [7, 8]. They are trusted by the communities. Therefore, involving them in health promotion can be more feasible than involving external resources [8, 9].They can raise awareness about the health problems more efficiently. It can also help improve the demand for health care in the communities. Improved awareness and health-seeking behaviour can eventually improve overall health outcomes [7–11].

Nepal is a low-income country, which experienced a major earthquake in April 2015, killing 9,000 people and affecting 2 million women of reproductive age, including 126,000 pregnant women [12, 13]. The immediate international response after the earthquake primarily focused on rescue and relief. However, evidence remains limited about the long-term recovery and health promotion for improving MNCH.

### Intervention

This study examines the effect of the community-based health promotion intervention. The intervention focused on improving the long-term health status of people affected by the earthquake. A local non-governmental organization (NGO), Green Tara Nepal (GTN) implemented a “Post Disaster Health Promotion Project” with the support from various international NGOs and academic institutions [14]. The intervention aimed at rebuilding the community health system through a participatory approach involving the local stakeholders and resources. GTN mobilized the local health promoters and female community health volunteers (FCHV) to improve the access to health care for mothers affected by the earthquake. GTN provided support in 7 earthquake affected village development committees (VDCs) of Dhading district; namely, Gumdi, Phulkharka, Dhuwakot, Khalte, Sunaulabazaar, Bhumesthan, and Tasarpu. The primary beneficiary was the mothers for the intervention activities with children below 12 months of age.

The preparatory phase for the intervention started in July 2016 and the key intervention activities ran between October 2016 and April 2018. GTN appointed one local health promoter (a nurse, health assistant or auxiliary nursing midwife) for each VDC and trained them using a standard curriculum. The curriculum was developed by GTN, which has been used successfully in the past project activities. The local health promoters led the community-level engagement activities for mothers to improve their knowledge and behaviour related to MNCH. The key intervention activities included: (a) capacity building of FCHV; (b) mothers’ group meetings; and (c) health system strengthening.

- Capacity building of FCHV

The health promoters organized meetings with the FCHVs and built their capacity as the facilitators to educate and empower the mothers’ groups in the communities. A total of 63 FCHVs from 7 VDCs attended the meetings regularly.

- Mothers’ group meetings

The FCHVs then worked closely with the health promoters to organize the meetings with the mothers’ groups and others such as mothers-in-law and male members of the family. These meetings focused on improving the knowledge and behaviours of mothers around seeking timely care during pregnancy and childbirth. The FCHVs conducted mother’s group meeting every month. The health promoters and FCHVs also made follow-up visits to households to discuss with mothers on MNCH-related health-seeking behaviour and to support mothers to access the recommended services. Mothers’ group meetings ran in all 63 wards in its catchment area throughout the intervention period.

In the meetings, FCHVs discussed the danger signs of pregnancy, childbirth, and in newborns. The danger signs of pregnancy and childbirth are based on the guidelines for Birth Preparedness Package [15]. The danger signs in newborns are based on the guidelines on Community Based Integrated Management of Newborn and Childhood Illness [16].

The danger signs are the key signs that could be easily recognized by the mothers to seek appropriate health care and prevent complications. The key danger signs of pregnancy include blurred vision, severe headaches, convulsions/fainting, swelling, and vaginal bleeding. The common danger signs of childbirth include labour longer than 8 hours, excessive vaginal bleeding, convulsion, and hand, foot or cord appearing first during vaginal delivery. The danger signs in newborns include fever, hypothermia, difficulties in breastfeeding, fast breathing, severe chest in-drawing, drowsiness or loss of consciousness, skin pustules, and cord discharge [17].

- **Health system strengthening**

GTN contributed in local health system strengthening by facilitating the coordination between health promoters and the local health facilities. GTN also helped health promoters to coordinate with the Health Facility Operation and Management Committees, VDCs, Ward Citizenship Forums, Women’s Information Centers and other organization working in health sectors. Additionally, the health promoters participated in the outreach immunization clinics of the local health facilities to motivate mothers to seek timely health care.

## Methods

### Study design and settings

This study is an uncontrolled before-and-after-study [18]. This study design measures the outcomes before and after the intervention in the same study sites without having separate control groups. Such a study design assumes that the observed differences between the pre- and post-interventions are due to the effect of the intervention. It is a type of quasi-experimental study design which is conducted when randomized control trials or having control groups are not feasible due to practical and ethical barriers [18].

This study was conducted in 7 earthquake-affected VDCs in Dhading district, Nepal. Dhading was one of the severely affected districts by the 2015 earthquake in Nepal. It lies adjacent to Gorkha district which was the epicentre for the earthquake [12]. The 2015 earthquake had destroyed 90% of houses in 21 VDCs of Dhading district and damaged 28 out of 52 health facilities interrupting the MNCH services [12]. We selected only 7 of the 21 VDCs in this study as they were the implementing sites of GTN. We did not choose other VDCs to avoid duplicating activities that could have been implemented by other organizations working in those areas.

### Study participants

The target population comprised mothers of reproductive age and living with a child below 12 months of age at the time of data collection. We recruited the mothers matching the eligibility criteria for the pre-intervention and post-intervention phases separately. We obtained the list of the mothers with a child younger than 12 months from the local health facilities.

### Sample size

We calculated the minimum required sample size using the formula to detect a difference between two proportions [19]. We used the baseline value of institutional delivery in Dhading after the earthquake to calculate the sample size. In 2015, the institutional delivery was just 33% in Dhading [14]. We expected the intervention to improve the proportion of institutional delivery by at least 12%. As a result, we calculated the minimum required sample size to be 256 for each group to detect a significant difference with a 95% confidence level and 80% power. However, we anticipated incomplete responses and thus extrapolated the sample size to 380 for each group.

In order to represent all the selected 7 VDCs proportionately, the research assistants attempted to collect 35% of the total population of each VDC through convenient sampling methods. As a result, the team collected complete data of 364 mothers in the pre-intervention group and 377 in the post-intervention group.

### Study variables

We adapted the study questionnaire from the baseline survey of the Community-Based MNCH Care Package of Nepal Family Health Program and Nepal Demographic and Health Survey [20, 21]. The questionnaire had closed-ended questions that comprised the questions on sociodemographic information of the mothers and their knowledge and behaviours related to MNCH.

The dependent variables included MNCH-related knowledge and behaviours [20]. We assessed the knowledge of danger signs of pregnancy, childbirth, and in newborns. We measured 5 key danger signs of pregnancy, which included the knowledge on blurred vision, severe headaches, convulsions/fainting, swelling, and vaginal bleeding.

We measured knowledge of 6 key danger signs of childbirth, which included labour longer than 8 hours, excessive vaginal bleeding, convulsion, and hand, foot or cord appearing first during vaginal delivery.

For the knowledge on danger signs in newborns, we measured knowledge of 8 danger signs which included fever, poor breastfeeding, fast breathing, chest in-drawing, drowsiness or loss of consciousness, skin infection around umbilicus, cord discharge, and chills or rigors.

Based on the Community-Based MNCH Care Package of Nepal Family Health Program, we considered knowledge of at least 3 danger signs each in pregnancy and childbirth, and 5 danger signs in newborns to be acceptable levels of knowledge [20]. The MNCH related behaviours included having at least one antenatal care (ANC) visit, having a minimum of four ANC visits, and institutional delivery [20].

The independent variables included socio-demographics of mothers such as age, education, ethnicity, religion, husband’s education, and wealth index. We calculated the wealth index score based on the mothers’ housing characteristics, household assets, and ownership of land and live stocks. We then divided the total wealth index score into the four categories: richer, rich, poor and poorer.

### Data collection

The data collection for this study took place before and after the intervention. The research assistants collected the data before the intervention in August and September 2016. They then collected the data after the intervention in May 2018. We first trained our research assistants on data collection and research ethics. The trained research assistants then consulted the FCHV and also used the immunization list from the health facilities to identify the households with children below 12 months of age. The research assistants then visited the houses of relevant mothers for the data collection. The same process was repeated for the data collection after the intervention. It took around 30 to 45 minutes to complete the questionnaire.

### Ethical considerations

We obtained the approval from Nepal Health Research Council. The research assistants obtained written informed consent from all participants. They also ensured their privacy and confidentiality during data collection.

### Data analysis

We analysed the data using chi-squared tests and independent sample t-tests to assess the difference in the general characteristics between the mothers recruited before the intervention and after the intervention. We then performed multivariable analysis using logistic regression models to examine the factors associated with knowledge and behaviours related to MNCH. We used SPSS version 23 with a *p*-value of less than 0.05 as the significance level for all analytical procedures.

## Results

### Characteristic of mothers in the pre-intervention and post-intervention groups

The pre-intervention group comprised 364 mothers and the post-intervention group 377. No major differences were detected in the general characteristics between both groups. The mean age was almost 24 years in both groups. Around 25% of the mothers had obtained primary education and around 50% had obtained secondary or higher level of education in both the groups. The level of education amongst the husbands of these mothers was also similar between both groups with around 30% of husbands having primary education and over 50% secondary or higher education. Over 70% of mothers followed Hinduism and around 20% were Buddhists in both groups. Most mothers belonged to *Janajati* ethnicity for both, with 65.7% in the pre-intervention group and 54.6% in the post-intervention group. The post-intervention group had richer mothers (27.3%) as compared the pre-intervention group (2.2%, P<0.001). (Table 1)

**Table 1:** Characteristics of mothers in pre- and post-intervention.

	Pre-intervention N = 364	Post-intervention N = 377	
	n (%)	n (%)	P-value
**Age (in years), mean (SD)**	24.4 (5.3)	24.7 (5.2)	0.535
**Education of mother**			
Illiterate	96 (26.4)	71 (18.8)	0.014
Primary education	90 (24.7)	97 (25.7)	0.753
Secondary and higher	177 (48.6)	199 (53.8)	0.258
**Husband's education**			
No education	44 (12.1)	40 (10.6)	0.526
Primary education	115 (31.6)	111 (29.4)	0.525
Secondary and higher	199 (54.7)	216 (57.3)	0.472
socioeconomic status			
**Ethnicity**			
Brahmin/Chetri	71 (19.5)	89 (23.6)	0.175
Dalit	54 (14.8)	78 (20.7)	0.037
Janajati	239 (65.7)	206 (54.6)	0.002
**Religion**			
Hindu	270 (74.2)	266 (70.6)	0.271
Buddhist	81 (22.3)	84 (22.3)	0.993
Others	13 (3.6)	27 (7.2)	0.031
**Wealth index**			
Richer	8 (2.2)	103 (27.3)	<0.001
Rich	103 (28.5)	140 (37.1)	0.012
Poor	88 (24.3)	71 (18.8)	0.07
Poorer	163 (45)	63 (16.7)	<0.001

### Knowledge

Table 2 shows the knowledge of danger signs during pregnancy, childbirth, and in newborns among the mothers from both groups. A higher proportion of mothers in the post-intervention group had knowledge of all the 5 danger signs of pregnancy as compared to those in the pre-intervention group. The commonest danger signs of pregnancy identified by the mothers were severe headache in both the pre-intervention group (70.3%) and post-intervention group (82.3%). The highest absolute difference was observed for the knowledge on vaginal bleeding (15.4%) between the pre-intervention group (52.7%) and post-intervention group (68.1%, p<0.001). The proportion of mothers having knowledge of at least three danger signs of pregnancy was also significantly higher in the post-intervention group (79.4%) than the pre-intervention group (59.1%, p <0.001).

**Table 2:** Mothers’ knowledge pre-intervention/post-intervention groups of danger signs during pregnancy, childbirth, and in newborns.

	Pre-intervention N = 364	Post-intervention N = 377	^b^Diff	
Knowledge	n (%)	n (%)	%	P-value
**Knowledge of** ^**a**^**DS in pregnancy**				
Blurred vision	252 (69.2)	265 (71.0)	1.8	0.116
Severe headache	256 (70.3)	307 (82.3)	12.0	0.001
Convulsions/fainting	206 (56.6)	221 (59.2)	2.6	0.63
Swelling	208 (57.1)	259 (69.4)	12.3	<0.001
Vaginal bleeding	192 (52.7)	254 (68.1)	15.4	<0.001
**Knowledge of at least 3 DS in pregnancy**	215 (59.1)	258 (79.4)	20.3	<0.001
**Knowledge of DS in childbirth**				
Labour longer than 8 hours	226 (80.1)	247 (65.7)	-14.4	<0.001
Hand appears first	177 (60)	254 (67.6)	7.6	<0.001
Foot appears first	162 (60.9)	256 (68.1)	7.2	<0.001
Cord appears first	149 (57.3)	243 (64.6)	7.3	<0.001
Excessive bleeding	154 (58.7)	224 (59.6)	0.9	<0.001
Convulsion	128 (53.3)	203 (54.0)	0.7	<0.001
**Knowledge of at least 3 DS in childbirth**	153 (42.0)	254 (67.4)	25.4	<0.001
**Knowledge of DS in newborns**				
Fever	302 (83.0)	294 (78.2)	-4.8	0.22
Poor breastfeeding	278 (76.4)	306 (81.4)	5	0.084
Fast breathing	257 (70.6)	288 (76.6)	6	0.009
Chest in drawing	227 (62.5)	249 (66.4)	3.9	<0.001
Drowsy/loss of consciousness	164 (45.3)	221 (58.8)	13.5	<0.001
Skin infection around umbilicus	152 (41.8)	205 (54.4)	12.6	<0.001
Cord discharge	178 (48.9)	230 (61.2)	12.3	<0.001
Chills/rigors	161 (44.2)	219 (58.2)	14.0	<0.001
**Knowledge of at least 5 DS**	178 (48.9)	219 (58.1)	9.2	0.012

^a^DS = Danger Signs

^b^Diff = Absolute difference between pre-intervention and post-intervention group

The proportion of mothers having knowledge of most of the six danger signs of childbirth was also higher for mothers in the post-intervention group than the pre-intervention group. However, the knowledge that labour longer than eight hours is considered as a danger sign was lower among the mothers in the post-intervention group (65.7%) than in the pre-intervention group (80.1%, p<0.001) with the largest absolute difference of 14.4%. The most common danger sign of childbirth identified by the mothers was labour longer than 8 hours in the pre-intervention group (80.1%) and foot appearing first in vaginal delivery in the post-intervention group (68.1%). The proportion of mothers knowing at least three danger signs of childbirth was significantly higher among the mothers from the post-intervention group (67.4%) than in the pre-intervention group (42.0%, p<0.001).

The knowledge about danger signs among newborns was also higher in the post-intervention group for majority of the questions. The commonest danger sign in the newborn was fever in the pre-intervention group (83%) and post-intervention group (78.2%). The highest absolute difference was observed on the knowledge on chill and rigors (14.0%) between the pre-intervention group (44.2%) and post-intervention group (58.2%, p<0.001).

The proportion of mothers having knowledge of at least five danger signs in the newborn was higher among the mothers from post-intervention group (58.1%) as compared to those from the pre-intervention group (48.9%, p = 0.012).

### MNCH health seeking behaviour

The proportion of mothers ever visiting health facility for ANC was higher in the post-intervention group (97.6%) than in the pre-intervention group (84.1%, P<0.001). The proportion of mothers having a minimum of four ANC visits was also higher among the mothers in the post-intervention group (87.6%) than pre-intervention group (51.6%, P<0.001). More mothers from the post-intervention group (83.0%) also had an institutional delivery than the pre-intervention group (63.2%, P<0.001). The highest absolute difference was observed for attending 4+ ANC (36%) between the pre-intervention group (51.6%) and post-intervention group (87.6%, p<0.001). Whereas, the absolute difference was 13.5% for ever attending ANC and 19.8% for institutional delivery. (Table 3)

**Table 3:** Health-seeking behaviours: ANC visits and institutional delivery.

	Pre-intervention	Post-intervention		
	N = 364	N = 377	^a^Diff	
	n (%)	n (%)	(%)	P-value
Ever attended ANC	306 (84.1)	368 (97.6)	13.5	<0.001
4+ ANC	188 (51.6)	326 (87.6)	36.0	<0.001
Institutional delivery	230 (63.2)	313 (83.0)	19.8	<0.001

^a^Diff = Absolute difference between pre-intervention and post-intervention group

### Factors associated with knowledge of danger signs

Table 4 shows the multivariable logistic regression models for knowledge of danger signs of pregnancy, childbirth and institutional delivery adjusted for the intervention group, mothers’ age, ethnicity, religion, education, and their husband’s education, and their wealth index. We used knowledge of at least 3 danger signs for pregnancy and childbirth, and 5 for newborns as the outcome variables in the logistic regression model.

**Table 4:** Factors associated with knowledge of danger signs.

	DS Pregnancy		DS childbirth		DS newborn	
	AOR	95% CI	AOR	95% CI	AOR	95% CI
**Intervention**						
Pre-intervention	1		1		1	
Post-intervention	2.87	1.90–4.36***	3.64	2.52–5.25***	1.57	1.09–2.25**
**Characteristics of mothers**						
Age	1.02	0.98–1.06	1.02	0.98–1.05	0.99	0.96–1.03
**Ethnicity**						
Brahmin/Chhetri	1		1		1	
Janajati	0.80	0.49–1.33	1.06	0.69–1.64	0.89	0.57–1.37
Dalit	1.56	0.81–3.01	1.99	1.10–3.31**	0.85	0.50–1.45
**Religion**						
Hindu	1		1		1	
Buddhist	0.39	0.25–0.62***	0.62	0.40–0.95**	0.57	0.37–0.87**
Others	0.29	0.11–0.52***	0.21	0.10–0.46***	0.36	0.17–0.77**
**Mother's Education**						
Illiterate	1		1		1	
Primary	2.30	1.32–3.99**	1.75	1.06–2.89	1.86	1.16–2.99
Secondary and higher	2.31	1.33–4.00**	2.21	1.34–3.65**	2.20	1.37–3.53**
**Husband's education**						
No education	1		1		1	
Primary education	1.47	0.82–2.61	1.63	0.93–2.86	1.49	0.82–2.40
Secondary and higher	2.28	1.25–4.16**	2.68	1.51–4.76**	1.73	1.07–2.96*
**Wealth index**						
Richer	1		1		1	
Rich	0.65	0.33–1.29	0.64	0.37–1.09	0.75	0.46-.1.24
Poor	0.71	0.34–1.52	0.75	0.41–1.37	0.85	0.49–1.50
Poorer	0.97	0.46–2.05	1.59	0.87–2.93	0.97	0.55–1.71
Parity						

DS = Danger signs

DS pregnancy (3 out of 5), DS childbirth (3 out of 6), and DS newborn (5 out of 8).

*P-value <0.05

**P-value<0.01

***P-value<0.001

The knowledge of danger signs of pregnancy increased by 3 folds (AOR = 2.9, P<0.001), childbirth by 4 folds (AOR = 3.8, P<0.001), and for newborns by 1.5 folds (AOR = 1.5, P<0.001) after the intervention.

As compared to mothers who followed Hinduism, Buddhists were less likely to have knowledge of danger signs of pregnancy (AOR = 0.39, P<0.001), danger signs of childbirth (AOR = 0.62, P = 0.029), and danger signs in newborns (AOR = 0.57, P = 0.009). Mothers who followed other religions were also less likely to have knowledge of danger signs of pregnancy (AOR = 0.24, P<0.001), childbirth (AOR = 0.21, P<0.001) and in newborns (AOR = 0.36, P = 0.008).

The mothers who had a primary education were more likely to have knowledge of danger signs of pregnancy (AOR = 2.30, P<0.001) as compared to those without education. The mothers who had secondary education or higher were also more likely to have knowledge of danger signs of pregnancy (AOR = 2.28, P<0.001), danger signs of childbirth (2.20, P = 0.002) and danger signs in newborns (AOR = 2.19, P = 0.001). The mothers with husbands who had a secondary or higher education were also more likely to have knowledge of danger signs of pregnancy (AOR = 2.28, P = 0.007), childbirth (AOR = 2.68, P = 0.001) and danger signs in newborns (AOR = 1.73, P = 0.048).

### Factors associated with the health seeking behaviours for ANC and institutional delivery

Table 5 shows the factors associated with health seeking behaviours on ever attending ANC, attending a minimum of four ANC, and having institutional delivery. The odds of ever attending ANC increased by 7 folds (AOR = 7.18, P<0.001) and to attend a minimum of 4 ANC increased by 5 folds (AOR = 5.09, P<0.001). However, the institutional delivery increased only slightly by 2.5 folds. (AOR = 2.56, P<0.001).

**Table 5:** Factors associated with the health seeking behaviours for ANC and institutional delivery.

	Ever ANC		4+ANC		Institutional delivery	
	AOR	95% CI	AOR	95% CI	AOR	95% CI
**Intervention**						
Pre-intervention	1		1		1	
Post-intervention	7.18	3.47–16.89***	5.09	3.31–7.84***	2.56	1.72–3.89***
**Characteristics of mothers**						
Age	0.96	0.91–1.02	0.92	0.88–0.96***	1.00	0.97–1.04
**Ethnicity**						
Brahmin/Chetri	1		1		1	
Janajati	1.28	0.64–2.59	0.59	0.36–0.99*	0.93	0.58–1.59
Dalit	0.75	0.32–1.77	0.64	0.34–1.19	0.90	0.50–1.62
**Religion**						
Hindu	1		1		1	
Buddhist	1.00	0.48–2.10	1.63	0.99–2.69	1.40	0.87–2.27
Others	1.34	0.29–6.22	0.72	0.33–1.60	1.43	0.63–3.22
**Mothers’ Education**						
No education	1		1		1	
Primary	0.70	0.31–1.57	0.80	0.46–1.40	1.56	0.93–2.65
Secondary and higher	1.13	0.48–2.66	1.05	0.47–1.45	1.44	0.85–2.44
**Husbands’ education**						
Illiterate	1		1		1	
Primary education	2.07	0.84–5.94	1.72	0.94–3.12	2.13	1.24–3.66**
Secondary and higher	0.94	0.39–2.26	1.42	0.77–2.62	3.88	2.19–6.89***
**Wealth index**						
Richer	1		1		1	
Rich	0.42	0.09–1.94	0.51	0.21–1.22	0.49	0.23–1.03
Poor	0.74	0.14–3.78	0.32	0.13–0.80**	0.48	0.22–1.06
Poorer	0.495	0.101–2.425	0.17	0.07–0.42***	0.47	0.21–1.03

*P-value <0.05

**P-value<0.01

***P-value<0.001

Older mothers were less likely to attend ANC for a minimum of four ANC visits (AOR = 0.92, P<0.001). Mothers of Janajati ethnicity were also less likely to attend four ANC visits as compared to those from Brahmin/Chhetri ethnic groups (AOR = 0.59, P = 0.047).

Moreover, poor and poorer mothers were less likely to attend a minimum of four ANC visits (AOR = 0.32, P = 0.015 and AOR = 0.17, P<0.001, respectively).

The mothers with husbands having a primary education (AOR = 2.13, P = 0.007) and secondary level or higher education were more likely to have an institutional delivery (AOR = 3.88, P<0.001) than those having husbands without education.

## Discussion

In this study, the mothers from the post-intervention group were more likely to have better MNCH related knowledge, and were also more likely to attend ANC and have an institutional delivery. The findings are reassuring that the health promotion activities were beneficial to improve the MNCH related knowledge and behaviour among the mothers in Dhading district after the earthquake.

The mothers from the post-intervention group were more likely to have knowledge of danger signs of pregnancy and childbirth, and in newborns. Mothers' knowledge about the MNCH-related danger signs is known to influence their utilization of skilled care during childbirth [22]. It is also recognized as an important factor to prevent the first delay to seek emergency obstetric care among the mothers [23]. This study indicates the intervention has enabled mothers to be better informed to seek timely health care services for MNCH.

However, the mothers from the religious minority groups were less likely to have knowledge of MNCH related danger signs as compared to the mothers following Hinduism. In Nepal, more than 80% of the population is Hindu, followed by 9% Buddhists, 4.4% Muslim, 1.3% Christians, and remaining other religions [24]. The minority groups are often more vulnerable to complications and more likely to be deprived of health care in the aftermath of disasters [25].

The religious minority group in this study are a vulnerable population as they are at a higher risk for health problems because of their shared social characteristics that is different from the majority group [26]. The factors that can act as barriers among the religious minority groups could include their own religious belief on ways to seek help of their illnesses [27]. They could also be discriminated and marginalized by the majority group because of the differences in their religious and socio-cultural practices [28]. Moreover, no policy was available to recognize the rights of minority groups and it could have affected their equitable access to education and health care [28]. However, this study cannot conclude if discrimination was taking place. A more in-depth study is necessary to understand the context better.

Moreover, this study focused on a generic intervention targeting all the mothers and their children affected by the earthquake. Such an approach may have failed to address the individualized needs of a minority population [29–31]. Thus, more focused efforts are needed to reach out to the minority population. To improve the knowledge, we should reinforce the information and modify the process of sharing knowledge with the minority groups.

In this study, the mothers with higher level of education were more likely to have knowledge of MNCH-related danger signs. Education plays a crucial role in enabling women to take better health-related decisions for themselves [32]. Higher status of education among mothers has been identified as a strong indicator for using MNCH services in Nepal [33]. Studies from Ethiopia and Madagascar have also indicated positive association between higher level of education among mothers and better knowledge of danger signs related to MNCH [34, 35]. The findings of this study suggests additional supports are necessary to the less educated mothers.

This study shows that the intervention had an effect on the mothers’ health care seeking behaviour such as attending ANC, attending a minimum of four ANC and having institutional delivery. However, the odds were the highest for mothers attending at least one ANC (AOR = 7.18), lower for mothers having a minimum of four ANC (AOR = 5.09), and the lowest for institutional delivery (AOR = 2.56). The findings are in line with the trends of service utilization as reported in the most recent Nepal Demographic Health Survey 2016 [36]. In Nepal, the proportion of mothers attending at least one ANC remains the highest (84%) whereas the use of four ANC is lower (69%) and the institutional delivery remains the lowest (58%) [36]. Institutional delivery ensures that women have access to skilled birth attendance. It helps to recognize the danger signs timely, and provide appropriate and adequate treatment. For low resource settings, it is the most crucial step in preventing maternal and newborn morbidity and mortality [37]. The findings highlight that more efforts are needed to convince mothers to attend a minimum of four ANC and to have institutional delivery for better MNCH outcomes.

Various socio-demographic factors could have attributed to the differences in MNCH service utilization in this study. While the utilization of at least one ANC was not associated with any socio-demographic characteristics, the use of a minimum of four ANC and institutional delivery was significantly associated with certain characteristics. The mothers of older age were less likely to attend four ANC in this study. A recent study on trends in inequity of health service utilization in Nepal has suggested that the use of four ANC services was found to decrease with increasing age as well [33]. Moreover, this study shows that poorer mothers were less likely to attend four ANC as compared to their richer counterparts. The wealth inequality has also been identified as a significant barrier for service utilization in Nepal [33]. This study highlight that the health promotion programmes must address inequity by involving the vulnerable population in every step of their interventions.

This study shows that the husband’s higher education level was significantly associated with the mother’s knowledge of MNCH related danger signs and behaviour on having institutional delivery. The male involvement is widely known to improve the MNCH outcomes [38]. In most patriarchal societies, men are the decision makers in the family and have control over their family’s health-seeking behaviours [39]. However, men may not always have adequate knowledge about the right thing to do as they are rarely involved in the MNCH related programmes [40]. In this study too, the men were not the direct beneficiaries of the programme. However, the significant association between the husband’s education with the mother’s knowledge and behaviour outcomes indicate that future programmes must involve men across all education levels.

This study should be interpreted with three major limitations. First, this study lacks control groups before and after the intervention which may affect the interpretation of the results. However, this study was conducted in a post-disaster setting, where the intervention had to target the entire community affected. Second, the mothers in both groups are different populations. It was not possible to follow the same group of mothers after the intervention because we needed a separate population to assess their recent behaviours related to ANC and institutional delivery. However, the two groups were comparable as they had similar characteristics and other differences were controlled in the multivariable analysis. Third, this study did not measure other obstetric details of the mothers such as parity which could also have played an important role in affecting mother’s knowledge related to MNHCH. Despite the limitations, this study provides important information on the health promotion activities for MNCH in low resource post-disaster settings.

## Conclusion

Health promotion activities have helped the disaster-affected mothers in improving the knowledge and behaviour related to MNCH. However, future health promotion activities should more prioritize the vulnerable population as they tended to receive less benefit from the same intervention. The innovative approaches involving men could also prove beneficial to sustain the progress. Further follow up on the health promotion activities even after the project ends could provide a long-term perspective on sustainability.

## Supporting information

S1 Table Household survey questionnaire for women (English). (DOC)

S2 Table Household survey questionnaire for women (Nepali). (DOC)
