# Supplementary material for: Assessing knowledge and behavioural changes on maternal and newborn health among mothers following post-earthquake health promotion in Nepal
Source: PLoS One. 2019 Jul 25;14(7):e0220191. doi: 10.1371/journal.pone.0220191 (PMC6657877; doi:10.1371/journal.pone.0220191)
Supplement: S1 Table — (DOC) [file pone.0220191.s001.doc]

**Post-Disaster Health Promotion Project, Dhading**

**S1 Table Household Survey Questionnaire for Women (English)**

**Instructions to interviewer:**

- Ask the screening questionnaire to household head or other senior members in the family for confirming the availability of eligible respondents.
- Obtain informed consent to proceed the interview.
- If eligible respondent is unavailable on the day of visit, make a second visit on the day second.

**Instruction for data entry:**

- Please enter “77” for ‘Record not available’, “88” for ‘Not Applicable’, and “99” for ‘Don’t Know’.

| Form No. |  |  |  |  |
| --- | --- | --- | --- | --- |

| Date dd/mm/yy | ……./ ………/ ………. |
| --- | --- |
| District | 1. Dhading |
| Name of VDC |  |
| Ward No |  |
| Village name |  |
| Household No. |  |
| Name of the household head |  |
| Relationship of respondent to household head (if respondent is not household head) |  |

**SCREENING Question:**

| Do you have a child under 12 months? (if no, do not continue questionnaire) | 1. Yes  2. No |
| --- | --- |
| If yes, how old is your youngest child? | … months … Days |

**Section 1: Household and Socio-demographic information**

| SN | Questions | | Coding categories | | | | | CODE |
| --- | --- | --- | --- | --- | --- | --- | --- | --- |
| 1. 101 | In what month and year were you born? Write in B.S. | | Month [_ _ | _ _] Year [_ _ | _ _]  99. Don’t know month  999. Don’t know year | | | | | DOB101 |
| 1. 102 | How old are you? (compare and correct 1.1 and/or 1.2 if inconsistent) | | Age in com pleted years [__ | __]  99. Don’t know | | | | | AGE102 |
| 1. 103 | What is your caste or ethnicity? (Write caste in space provided. Do not fill box. Refer CBS code) | | ______________________  Caste/Ethnicity | | | | | ETH103 |
| 1. 104 | What is your religion? | | 1. Hindu  2. Buddhist  3. Muslim  4. Christian  5. Other (specify) ________________ | | | | | REL104 |
| 1. 105 | What is the highest class you completed? | | 1. None  2. Primary  3. Middle/LWS  4. Secondary/HS/TECH/VOC  5. Tertiary or above  6. Non-formal education  99. Don’t Know | | | | | EDU105 |
| 1. 106 | What is your occupation, that is, what kind of work do you mainly do? | | 1. Housewife  2. Labour  3. Agriculture  4. Private office employee  5. Govt. office employee  6. Small business – sewing, carpentry  7. Shopkeeper  9. Others (Specify) | | | | | OCC106 |
| 1. 107 | Age of husband (in completed years) | | Age in completed years [__ ]  99. Don’t know | | | | | HAG107 |
| 1. 108 | What is your husband’s level of education? - | | 1. None  2. Primary  3. Middle/LWS  4. Secondary/HS/TECH/VOC  5 Tertiary or above  6. Non-formal education  99. Don’t Know | | | | | HED108 |
| 1. 109 | What is your husband’s occupation? | | 1. Labour  2. Agriculture  3. Private office employee  4. Govt. office employee  5. Small business – sewing, carpentry  6. Shopkeeper  7. Unemployed  8. Student  9. Others (Specify) | | | | | HOC109 |
| 1. 110 | How many people live in your house? | | - Total ________ - Young People & Adults (age 10 or above)   _______   - Children (below 10 yrs) ­­_____ | | | | | FM110a  FM110b  FM110c |
| 1. 111 | Do you have own mobile phone? | | 1. Yes 2. No | | | | | MOB111 |
| 1. 112 | Where are you currently living? | | 1. In own home  2. In rented property  3. Living with relative  9. Other (specify) _____________ | | | | | LIVE112 |
| 1. 113 | Main material of the floor (Record observation) | | 1. Earth/mud/dung  2. Wood planks  3. Linoleum / carpet  4. Ceramic tiles, marble chips  5. Cement  9. Other (specify) | | | | | FLO113 |
| 1. 114 | Main material of the roof (Record observation) | | 1. Thatch  2. Metal  3. Tiles  4. Cement  5. Rock shingles  6. No roof  9. Other (specify) ______________ | | | | | ROF114 |
| 1. 115 | Main material of the walls (Record observation) | | 1. Bamboo with mud  2. Stone with mud  3. Adobe/bricks  4. Plywood  5. Unfinished wood  6. Stone with cement  7. Cement blocks  8. Wood planks  10. No walls  9. Other (specify) ______________ | | | | | WAL115 |
| 1. 116 2. # | Does your household have: | | | 1. YES | | 2.No | |  |
|  | 1. Electricity | | | 1 | | 2 | | EL116a |
|  | 1. Radio | | | 1 | | 2 | | RD116b |
|  | 1. Television | | | 1 | | 2 | | TV116c |
|  | 1. Telephone (Landline) | | | 1 | | 2 | | TL116d |
|  | 1. Refrigerator | | | 1 | | 2 | | RF116e |
|  | 1. Computer | | | 1 | | 2 | | CM116f |
|  | 1. Wall clock | | | 1 | | 2 | | WC116g |
|  | 1. Gas geyser | | | 1 | | 2 | | GG116h |
|  | 1. Solar panel | | | 1 | | 2 | | SP116i |
| 1. 117 2. ## | Does any member of your household own: | | | 1. YES | | 2.No | |  |
|  | Bicycle /Rickshaw | | | 1 | | 2 | | BC117a |
|  | Motorcycle/scooter | | | 1 | | 2 | | MC117b |
|  | Tempo | | | 1 | | 2 | | TM117c |
|  | Car/Truck ­ | | | 1 | | 2 | | CR117d |
|  | Cart (bull cart) | | |  | |  | | CT117e |
|  | Other (specify) ­­­_______________ | | |  | |  | | OT117f |
| 1. 118# | What is the main source of drinking water for members of your household? | 1. Piped water  2. Piped into house/yard/plot  3. Public / neighbor’s tap  4. Dug well/Well in house/yard/plot  5. Public/neighbor’s well  6. Tube well/borehole/ Tube well in yard/plot  7. Public/neighbor’s tube well  8. Surface water  10. Spring/kuwa  11. River/stream/pond/lake  12. Stone tap/dhara  9. Other (specify) ___________________ | | | | | | WAT118 |
| 1. 119 | What type of toilet facilities does your house have? | 1. Flush toilet  2 Traditional pit toilet  3. Ventilated improved pit latrine  4. No facility / bush / field  9. Other (specify) | | | | | | TOI119 |
| 1. 120 2. ## | If you have no toilet at your house, what is the reason? | 1. Lack of financial resources  2. Lack of time  3. No land -  4. Do not like defecating in toilet  5. Do not know importance of toilet  6. New house/still to make toilet  9. Other (specify) | | | | | | NTl120 |
| 1. 121 | Is there enough water in the toilet, where do you go? | | 1. Yes  2. No | | | | | WT121 |
| 1. 123 | Is anyone in your family currently working abroad? | | 1. Yes  2. No | | | | | ABR123 |
| 1. 124# | If yes, in which country/countries are they working? | | 1 ........................................  2......................................... | | | | | CNT122a  CNT122b |
| 1. 125 | Are they sending money to your household? | | 1. Yes  2. No  88. Not applicable (recently gone)  99. Do not know | | | | | NRS125 |
| 1. 126 2. # | What type of fuel does your household mainly use for cooking? (max 2 answers) | | | | Yes | | No |  |
|  | 1. Electricity | | | | 1 | | 2 | FL126a |
|  | 1. LPG (Gas) | | | | 1 | | 2 | FL126b |
|  | 1. Biogas | | | | 1 | | 2 | FL126c |
|  | 1. Kerosene | | | | 1 | | 2 | FL126d |
|  | 1. Wood | | | | 1 | | 2 | FL126e |
|  | 1. Animal dung | | | | 1 | | 2 | FL126f |
|  | 1. Other (specify) __________________ | | | | 1 | | 2 | FL126g |

# Multiple answers possible

**Antenatal Care and Seeking Care**

Note: these questions are related to the woman’s LAST pregnancy

Interviewer: “Now, I would like to ask you some questions about when you were pregnant prior to your most recent delivery.”

| Q. # | Question | | Codes | | | | | | | |  |
| --- | --- | --- | --- | --- | --- | --- | --- | --- | --- | --- | --- |
| 1. 403 | Did you see anyone for antenatal care for the pregnancy prior to your most recent delivery? | | 1. Yes  2. No | | | | | | | | ANC403 |
| 1. 404 # | Whom did you see?  (Ask: “Anybody else?” Continue until no further answers. Circle all responses.) | | 1. Doctor  2. Nurse/ANM/MCHW  3. HA/AHW/VHW 4. FCHV  5. TBA  9. Other (specify) | | | | | | | | ANC404 |
| 1. 405 | How many times did you have antenatal check ups during your last pregnancy? | | | # of times …….  99. Don’t know | | | | | | | ANC405 |
| 1. 406 | At which gestational week did you receive ANC? | ……… Month or……… weeks | | | | | | | Don’t remember | |  |
|  | 1. 1st ANC | ……… Month or……… weeks | | | | | | | 99 | | ANC406a |
|  | 1. 2nd ANC | ……… Month or……… weeks | | | | | | | 99 | | ANC406b |
|  | 1. 3rd ANC | ……… Month or……… weeks | | | | | | | 99 | | ANC406c |
|  | 1. 4th ANC | ……… Month or……… weeks | | | | | | | 99 | | ANC406d |
|  | 1. 5th ANC | ……… Month or……… weeks | | | | | | | 99 | | ANC406e |
|  | 1. 6th ANC | ……… Month or……… weeks | | | | | | | 99 | | ANC406f |
|  | 1. 7th ANC | ……… Month or……… weeks | | | | | | | 99 | | ANC406g |
|  | 1. 8th ANC | ……… Month or……… weeks | | | | | | | 99 | | ANC406h |
|  | 1. 9th ANC | ……… Month or……… weeks | | | | | | | 99 | | ANC406i |
|  | 1. 10th ANC | ……… Month or……… weeks | | | | | | | 99 | | ANC406j |
| 1. 407 | Check if complete 4 ANC as per the schedule | | | 1. Yes  2. No  77. Record not available ­ | | | | | | |  |
| 1. 408 | Did you receive maternal health record book in the last pregnancy? | | | 1. Yes  2. No  99. Don’t know | | | | | | | ANC408 |
| 1. 409 | Do you have an antenatal card from when you were pregnant prior to your last delivery? | | | 1. Yes (seen)  2. Yes (not seen)  3. No | | | | | | | ANC409 |
| 1. 411# | As part of your antenatal care during the pregnancy prior to your most recent delivery, were any of the following done at least once?  (Read each service, circle appropriate response.) | | | | Yes | | No | | | Don’t know |  |
|  | 1. Was your abdomen examined? | | | | 1 | | 2 | | | 99 | ANC411a |
|  | 1. Did you receive iron tablets? | | | | 1 | | 2 | | | 99 | ANC411b |
|  | 1. Did you receive deworming tablets? | | | | 1 | | 2 | | | 99 | ANC411c |
|  | 1. Was your weight measured? | | | | 1 | | 2 | | | 99 | ANC411d |
|  | 1. Was your height measured? | | | | 1 | | 2 | | | 99 | ANC411e |
|  | 1. Was your blood pressure measured? | | | | 1 | | 2 | | | 99 | ANC411f |
|  | 1. Did you give a urine sample? | | | | 1 | | 2 | | | 99 | ANC411g |
|  | 1. Did you give a blood sample? | | | | 1 | | 2 | | | 99 | ANC411h |
| 1. 414 | Did you receive tetanus toxoid injection when you were pregnant prior to your most recent delivery? | | 1. Yes  2. No  99. Don’t know | | | | | | | | ANC414 |
| 1. 415 | Instructions for interviewer:  (Look at the antenatal card and record the number of TT injections for which a date is listed on the card during her last pregnancy.) | | Number of shots _ □□  77. Card not available  99. Don’t know | | | | | | | | ANC415 |
| 1. 416 | Were you given or did you buy any iron/folic acid tablets when you were pregnant prior to your most recent delivery? (SHOW IRON TABLETS.) | | 1. Yes  2. No  99. Don’t know | | | | | | | | ANC416 |
| 1. 419 | When you were pregnant, did you receive deworming tablets? | | 1. Yes  2. No  99. Don’t know | | | | | | | | ANC419 |
| 1. 424 | Who decided that you would go for your antenatal check-up? | | 1. Myself  2. Husband  3. Mother-in-law  4. Jointly  9. Other (specify) | | | | | | | | ANC424 |
| 1. 425 | How many check-ups should a woman have with a trained health worker while she is pregnant?_ | | # of checkups ___________  99. Don’t know | | | | | | | | ANC425 |
| 1. 426# | What are the signs/symptoms during pregnancy indicating the need to seek immediate care?  (Probe: “Any other?”)  (Circle all responses.) | | 1. Blurred vision  2. Severe headache  3. Convulsion, fainting  4. Swelling of hands, body or face  5. Any amount of vaginal bleeding | | | | | | | | ANC426a  ANC426b  ANC426c  ANC426d  ANC426e |
| 1. 427# | When you were pregnant, did you experience any of the following problems at anytime? (Read out all responses one after another. Record all responses accordingly). | | | | | Yes | | No | | Don’t know |  |
|  | 1. Blurred vision? | | | | | 1 | | 2 | | 99 | PRB427a |
|  | 1. Severe lower abdominal pain? | | | | | 1 | | 2 | | 99 | PRB427b |
|  | 1. Severe headache? | | | | | 1 | | 2 | | 99 | PRB427c |
|  | 1. Convulsion, loss of consciousness? | | | | | 1 | | 2 | | 99 | PRB427d |
|  | 1. Swelling of the hands, body or face? | | | | | 1 | | 2 | | 99 | PRB427e |
|  | 1. Any vaginal spotting or bleeding? | | | | | 1 | | 2 | | 99 | PRB427f |
| 1. 428# | What did you do or whom did you consult for this problem? (Prompt: “anything else?” Circle all answers) | | | | | Yes | | No | | Don’t know |  |
|  | 1. Traditional treatment at home | | | | | 1 | | 2 | | 99 | SOL428a |
|  | 1. Given medicine at home | | | | | 1 | | 2 | | 99 | SOL428b |
|  | 1. Hospital | | | | | 1 | | 2 | | 99 | SOL428c |
|  | 1. PHCC /HP/ SHP | | | | | 1 | | 2 | | 99 | SOL428d |
|  | 1. Pvt. Clinic/Nursing Home | | | | | 1 | | 2 | | 99 | SOL428e |
|  | 1. Bought medicine from pharmacy | | | | | 1 | | 2 | | 99 | SOL428f |
|  | 1. Consulted FCHV | | | | | 1 | | 2 | | 99 | SOL428g |
|  | 1. Consulted Dhami /Jhankri | | | | | 1 | | 2 | | 99 | SOL428k |
|  | 1. Consulted relative/neighbor/friend | | | | | 1 | | 2 | | 99 | SOL428l |
|  | 1. Nothing | | | | | 1 | | 2 | | 99 | SOL428m |
|  | 1. Other: ______________________ | | | | | 1 | | 2 | | 99 | SOL428n |
| 1. 429 | How did you know about ANC checkups? | | | | | Yes | | No | | Don’t know |  |
|  | 1. From Family members | | | | | 1 | | 2 | | 99 | INF429a |
|  | 1. From Radio/TV | | | | | 1 | | 2 | | 99 | INF429b |
|  | 1. Health workers | | | | | 1 | | 2 | | 99 | INF429c |
|  | 1. Friends/relatives / Neighbour/community people | | | | | 1 | | 2 | | 99 | INF429d |
|  | 1. School/college/teacher | | | | | 1 | | 2 | | 99 | INF429e |
|  | 1. Female community health volunteer | | | | | 1 | | 2 | | 99 | INF429f |
|  | 1. Other | | | | | 1 | | 2 | | 99 | INF429g |

# Multiple answers possible

**Delivery Care**

**Interviewer: “Now, I would like to ask you some questions about your most recent delivery.”**

| Q. # | Question | | | Codes | | | |  |
| --- | --- | --- | --- | --- | --- | --- | --- | --- |
| 1. 501 | Where did you give birth in your most recent delivery? | | | 1. Hospital  2. PHCC  3. Health post  4. Pvt. Clinic/n. Home  5. home  6. On the way to health facility  9. Other (specify) ______________ | | | | DEL501 |
| 1. 502# | Who assisted with your most recent delivery?  (Prompt: “Anybody else?”)  (Circle all responses) | | | 1. Doctor  2. Nurse/ANM  3. HA/AHW  4. FCHV  5. TBA  6. Friends/Neighbors  7. Mother-in-law  8. Nobody  10. Maternal members (mother, sisters)  9. Other (specify) ________________  99. Don’t know | | | | DEL502 |
| 1. 504 | Was your child delivered by caesarean section?  (Prompt: Ask “did a doctor cut open your abdomen to deliver the baby?”) | | | | 1. Yes  2. No | | | DEL504 |
| 1. 506 | Who was the main person who decided where your most recent delivery would take place? | | 1. Self  2. Mother-in-law  3. Father-in-law  4. Husband  5. Mother / father  6. Other relative  9. Other (specify) ________________  99. Don’t know | | | | | DEC506 |
| 1. 507# | What are the signs/symptoms during labor indicating the need to seek immediate care?  (Probe: “Any other?”)  (Circle all responses.) | | 1. Labor longer than 8 hours  2. Appearance of baby’s hand first  3. Appearance of baby’s leg first  4. Appearance of umbilical cord first  5. Excessive bleeding before or after delivery  6. Convulsion  9. Other (specify) ________________  99. Don’t know | | | | | INF507a  INF507b  INF507c  INF507d  INF507e  INF507f  INF507g  INF507h |
| 1. 508 | During your delivery, did you experience any of the following problems at anytime? (Read out all responses one after another.) (Record all responses accordingly). | | | | | Yes | No |  |
|  | 1. So much bleeding that it wet your clothes and you feared it was life threatening | | | | | 1 | 2 | PRB508a |
|  | 1. High fever | | | | | 1 | 2 | PRB508a |
|  | 1. Waters ruptured for more than 24 hours with no delivery | | | | | 1 | 2 | PRB508b |
|  | 1. Convulsions | | | | | 1 | 2 | PRB508c |
|  | 1. Severe headache | | | | |  |  | PRB508d |
|  | 1. Prolonged labor (>8 hours) | | | | | 1 | 2 | PRB508e |
|  | 1. Malpresentation | | | | | 1 | 2 | PRB508f |
|  | 1. Retained placenta | | | | | 1 | 2 | PRB508g |
| 1. 509# | What did you do or whom did you consult for this problem? (Prompt: “anything else?”. Circle all answers) | 1. Traditional treatment at home  2. Given medicine at home  3. Hospital  4. PHCC /HP/ SHP  5. Pvt. Clinic/n. Home  6. Consulted FCHV  7. Consulted HW  8. Consulted dhami / jhankri  10. Consulted relative/neighbor/friend  11. Nothing  9. Other(specify) ________________ | | | | | | SOL509 |

# Multiple answers possible

| 1. 614 | During the six weeks following your most recent delivery, did you experience any of the following problems at anytime? (Read out all responses one after another. ) (Record all responses accordingly.) | | Yes | No | Don’t know |  |
| --- | --- | --- | --- | --- | --- | --- |
|  | 1. Heavy bleeding and you feared it was life threatening | | 1 | 2 | 99 | DS614a |
|  | 1. High fever | | 1 | 2 | 99 | DS614b |
|  | 1. Severe lower abdominal pain | | 1 | 2 | 99 | DS614c |
|  | 1. Convulsions | | 1 | 2 | 99 | DS614d |
|  | 1. Severe headache | | 1 | 2 | 99 | DS614e |
|  | 1. Swelling of the hands, body or face | | 1 | 2 | 99 | DS614f |
|  | 1. Excessive fatigue | | 1 | 2 | 99 | DS614g |
|  | 1. Bad smelling vaginal discharge | | 1 | 2 | 99 | DS614h |
|  | 1. Breast complications | | 1 | 2 | 99 | DS614i |
| 1. 615 | Where did you go? | 1. Hospital  2. PHCC  3. Health Post  4. Pvt. Clinic/n. Home  5. Pharmacy  6. Did nothing  9. Other (specify) ________________  99. Don’t know/ Don’t remember | | | | REF615 |

# Multiple answers possible

**Neonatal Care**

**Interviewer: “Now, I would like to ask you some questions about the health of your child during the month after your most recent delivery.”**

| Q. # | Question | | Codes |  |
| --- | --- | --- | --- | --- |
| 1. 701 | Interviewer: Check question Q4 and confirm it was a live birth | | 1. Yes  2. No  99. Don’t know | LIV701 |
| 1. 702 | Was a Clean Home Delivery Kit used during delivery? (Show example of a CHDK) | | 1. Yes  2. No  99. Don’t know | CDK702 |
| 1. 703 | What instrument was used to cut the cord? | | 1. New Blade  2. Boiled Blade  3. Unboiled used blade  4. Knife  5. Grass Cutter (hansiya)  6. Weapon (khukuri)  7. Scissors  8. Born in hospital  9. Other (specify)  99. Don’t know | NNC703 |
| 1. 704 | Was the cord cut before the placenta was delivered? | | 1. Yes  2. No  99. Don’t know | NNC704 |
| 1. 705 | What was used to tie the cord? | | 1. New ties  2. Boiled string or thread  3. Unboiled used thread  4. Clip  9. Other (specify)  99. Don’t know | NNC705 |
| 1. 706 | Did anybody apply anything on the stump after the baby’s cord was cut? | | 1. Yes  2. No  99. Don’t know | NNC706 |
| 1. 707# | What did they apply? (Prompt: “Anything else?”) (Circle all responses). | | 1. Chlorhexidine Navi Malham  2. Oil  3. Ash  4. Sindoor  5. Ointment/powder  6. Animal dung  7. Turmeric/turmeric powder  8. Ghyu  9. Other (specify)  99. Don’t know | NNC707 |
| 1. 708 | Was your baby wrapped in cloth before the placenta was delivered? | | 1. Yes  2. No  99. Don’t know | NNC708 |
| 1. 709 | How long after birth was your baby bathed for the first time? | | 1. Within 1 hour  2. 2-24 hours  3. After 24 hours  99. Don’t know | NNC709 |
| 1. 710 | How long after birth did you first put to the breast? | | 1. During the first hour after delivery  2. More than 1 hour  9. Other (specify)  99. Don’t know | NNC710 |
| 1. 711 | Did you give the first liquid (begauti) that came from your breasts? | | 1. Yes  2. No  99. Don’t know | NNC711 |
| 1. 712 | Are you still breastfeeding? | | 1. Yes  2. No | NNC712 |
| 1. 713 | Please tell me when should a newborn child be bathed after the birth? | 1. Immediately after the birth  2. Within 24 hours after birth  3. After 24 hours after birth  4. Should not be bathed  9. Other (specify)  99. Don’t know | | NNC713 |
| 1. 714 | Please tell me when should a newborn child be breast fed for the first time after the birth? | 1. Immediately after the birth  2. After the placenta is out  3. After bathing the new born  4. After 24 hours after birth  9. Other (specify) _________________  99. Don’t know | | NNC714 |

| 1. 720 | What are the danger signs of newborn those needs immediate referrals to health facility for the treatment? | Yes | No | Don’t know |  |
| --- | --- | --- | --- | --- | --- |
|  | 1. Fever | 1 | 2 | 99 | DS720a |
|  | 1. Feeding problem | 1 | 2 | 99 | DS720b |
|  | 1. Fast breathing | 1 | 2 | 99 |  |
|  | 1. Chest-in-drawing – | 1 | 2 | 99 | DS720d |
|  | 1. Drowsy / Unconscious | 1 | 2 | 99 | DS720e |
|  | 1. Skin pustules | 1 | 2 | 99 | DS720f |
|  | 1. Discharge from cord | 1 | 2 | 99 | DS720g |
|  | 1. Felt cold | 1 | 2 | 99 | DS720h |
| 1. 721 | Did your newborn experience any of the following health problems at anytime in the first month following delivery? **(Record all responses)** | Yes | No | Don’t know |  |
|  | 1. Fever | 1 | 2 | 99 | PRB721a |
|  | 1. Feeding problem | 1 | 2 | 99 | PRB721b |
|  | 1. Fast breathing | 1 | 2 | 99 | PRB721c |
|  | 1. Chest-in-drawing | 1 | 2 | 99 | PRB721d |
|  | 1. Drowsy / Unconscious | 1 | 2 | 99 | PRB721e |
|  | 1. Skin pustules | 1 | 2 | 99 | PRB721f |
|  | 1. Skin around cord red | 1 | 2 | 99 | PRB721g |
|  | 1. Felt cold | 1 | 2 | 99 | PRB721h |
| 1. 722# | What did you do or whom did you consult for this problem?  (Prompt: “anything else?” Circle all answers). | Yes | No | Don’t know |  |
|  | 1. Traditional treatment at home | 1 | 2 | 99 | SOL722a |
|  | 1. Given medicine at home | 1 | 2 | 99 | SOL722b |
|  | 1. Hospital | 1 | 2 | 99 | SOL723c |
|  | 1. PHCC/HP/SHP | 1 | 2 | 99 | SOL723d |
|  | 1. Pvt. Clinic/Nursing Home | 1 | 2 | 99 | SOL723e |
|  | 1. Bought medicine from pharmacy | 1 | 2 | 99 | SOL723f |
|  | 1. Consulted FCHV | 1 | 2 | 99 | SOL723g |
|  | 1. Consulted Traditional healer | 1 | 2 | 99 | SOL723h |
|  | 1. Consulted relative/neighbor/friend | 1 | 2 | 99 | SOL723i |
|  | 1. Nothing | 1 | 2 | 99 | SOL723j |
|  | 1. Other (specify) _____________________ | 1 | 2 | 99 | SOL723k |
