# Supplementary material for: Assessing knowledge and behavioural changes on maternal and newborn health among mothers following post-earthquake health promotion in Nepal
Source: PLoS One. 2019 Jul 25;14(7):e0220191. doi: 10.1371/journal.pone.0220191 (PMC6657877; doi:10.1371/journal.pone.0220191)
Supplement: S2 Table — (DOC) [file pone.0220191.s002.doc]

**lu|g tf/f g]kfn (Green Tara Nepal)**

k|fs[lts k|sf]kkl5sf] :jf:Yo k|jw{g kl/of]hgf

**S2 Table Household Survey Questionnaire for women (Nepali)**

**! jif{ d"lgsf aRrf ePsf dlxnfx?nfO{ ;f]lwg] k|ZgfjnL**

**Instructions to interviewer:**

- To; kl/jf/df ev{/} aRrf hGdfPsL cfdf x'g'x'G5 eGg]s'/f kl/jf/sf ;b:ox?nfO{ ;f]w]/ ;'lglZrt ug'xf];\ . tkfOsf] 3/df !@ dlxgf eGbf sd pd]/sf] aRrf 5< -olb 5}g eg] k|Zg g;f]Wg'xf];\_ .
- olb 5 eg] d~h'/Lgfdf lng'xf];\ / cGtjf{tf z'? ug{'xf];\ .
- olb pQ/bftfl;t e]6 gePdf k'gM 3/e]6 u/L cGtjf{tf lng'xf];\ .

**Instruction for data entry:** lgb]{zgM

olb pQ/bftf e]6 gePdf æ&&” , pQ/ c;Dalwt ePdf æ**” / yfxf 5}g eg] æ((” n]Vg'xF];\_ .

| Form No. |  |  |  |  |
| --- | --- | --- | --- | --- |

| ldlt: -ut] . dlxgf . ;fn_ | ……./ ………/ ………. |
| --- | --- |
| lhNnf:……………………………………… |  |
| ;fljssf] uf= lj= ;=…………………… |  |
| ;fljssf] j8f g+= …………………………………… |  |
| ufp . 6f]n ………………………………… |  |
| 3/ g+= ……………………………… |  |
| 3/d"nLsf] gfd ……………… |  |
| 3/d"nLl;t pQ/bftfsf] gftf |  |

**SCREENING Question:**

| s] tkfOsf] !@ dlxgf eGbf sd pd]/sf] aRrf 5< | 1. -5_  2. -5}g_ |
| --- | --- |
| tkfOsf] ;aeGbf ;fgf] aRrf slt pd]/sf] 5< | -=== dlxgf ===== lbg_ |

**;fdflhs tyf kfl/jf/Ls ljj/0f**

| SN | Questions | | Coding categories | | | | | CODE |
| --- | --- | --- | --- | --- | --- | --- | --- | --- |
| 1. 101 | tkfOsf] hGd s'g dlxgf / ;fndf ePsf] xf]< la= ;+= df n]Vg'xf];\ . | | -dlxgf_ [_ _ | _ _] -;fn_ [_ _ | _ _]  99. -dlxgf yfxf 5}g_  999. -jif{ yfxf 5}g_ | | | | | DOB101 |
| 1. 102 | tkfOsf] pd]/ slt eof]< k'/f ePsf] pd]/ n]Vg'xf];\ . | | -k'/f ePsf] jif{_ [__ | __]  99. -yfxf 5}g_ | | | | | AGE102 |
| 1. 103 | tkfO{sf] hft s] xf] < ;a}sf] y/ CBS code cg';f/ hft n]Vg'xf];\ . | | ______________________  -hft. y/_ | | | | | ETH103 |
| 1. 104 | tkfO{ s'g wd{ dfGg'x'G5< | | 1. -lxGb'_  2. -a'4_  3 -d'l:nd_  4. -lqm:rLog_  5. O-cGo_ ________________ | | | | | REL104 |
| 1. 105 | tkfO{n] sltsIff;Dd k9\g'ePsf] 5< | | 1. -lg/If/_  2. -k|fylds tx_  3. -lgDg dfWoflds tx_  4. -dfWoflds . pRr dfWoflds tyf Joj;foLs tx_  5. -la P jf ;f] eGbf dfyL­­_  6. -cgf}krfl/s lzIff­­_  99. -yfxf gePsf]_ | | | | | EDU105 |
| 1. 106 | tkfO{sf] clxn]sf] d'Vo k]zf -sfd_ s] xf] < | | 1. -u[lx0fL_  2. -Hofnf dhb'/L_  3. -s[ifL_  4. -lghL sDkgLdf hflu/]_  5. -;/sf/L hflu/]_  6. -3/]n' pBf]u, l;nfO{, l;sdL{_  7. -k;n_  9. -cGo_______________ | | | | | OCC106 |
| 1. 107 | tkfO{sf] >Ldfg slt jif{sf] x'g'x'G5< | | -k'/f ePsf] jif{_ [__ ]  99. yfxf 5}g | | | | | HAG107 |
| 1. 108 | tkfO{sf] >Ldfg\n] slt;Dd k9\g' ePsf] 5< | | 1. -lg/If/_  2. -k|fylds tx_  3. -lgDg dfWoflds tx_  4. -dfWoflds . pRr dfWoflds tyf Joj;foLs tx_  5 -la P jf ;f] eGbf dfyL_  6. -cgf}krfl/s lzIff­­_  99. -yfxf gePsf_] | | | | | HED108 |
| 1. 109 | tkfO{sf] >Ldfg\sf] d'Vo k]zf s] xf] < | | 1. -Hofnf dhb'/L_  2. -s[ifL_  3. -lghL sDkgLdf hflu/]_  4. -;/sf/L hflu/]_  5. -3/]n' pBf]u, l;nfO{, l;sdL{_  6. -k;n­_  7. -a]/f]huf/­  8. -ljBfyL{_  9. -cGo_______________ | | | | | HOC109 |
| 1. 110 | tkfO{sf] 3/df slt hgf x'g'x'G5 < | | - -hDdf­_ ________ - -jo:s_ _______ - -aRrf !) jif{ d'lg_ ­­_____ | | | | | FM110a  FM110b  FM110c |
| 1. 111 | tkfO{sf] cfkm\g} df]jfOn kmf]g 5< | | 1. -5_ 2. -5}g_ | | | | | MOB111 |
| 1. 112 | tkfO{ clxn] sxf““ al; /xg' ePsf] 5 < | | 1. -cfkm\g} 3/df_  2. -ef8fdf_  3. -gft]bf/l;t al; /x]sf]_  9. -cGo_ _____________ | | | | | LIVE112 |
| 1. 113 | 3/sf] e'O{ s:tf] k|sf/sf]] 5<  -cjnf]sg_ | | 1. -uf]a/ . df6f]_  2. -sf7 . kfs]{l6ª_  3. -sfk]{6_  4. -6fon . dfa{n_  5. -l;d]G6_  9. -cGo________________ | | | | | FLO113 |
| 1. 114 | 5fgf s:tf] k|sf/sf] 5fgf] ePsf] 3/df a:g'ePsf] 5<  -cjnf]sg_ | | 1. -v/_  2. -h:tf_  3. -6fon_  4. -l;d]G6 9nfg_  5. -9'+uff_  6. -5fgf gePsf]_  9. -cGo_ ______________ | | | | | ROF114 |
| 1. 115 | s:tf] k|sf/sf] ufx|f] ePsf] 3/df a:g'ePsf] 5<  -cjnf]sg_ | | 1. -a“f;sf]_  2. -9'+ufsf]_  3. -O§f_  4. -KnfO{p8_  5. -sf7_  6. -l;d]G6 . 9'+uf_  7. -Ans_  8. -sf7sf] kmNofs_  10. -ufx|f] gePsf]_  9. -cGo_ ______________ | | | | | WAL115 |
| 1. 116 | -s] tkfO{sf] 3/df lgDg ;'ljwfx? 5g\ <_ | | | 1. -5_ | | 2. -5}g_ | |  |
|  | 1. -lah'nL_ | | | 1 | | 2 | | EL116a |
|  | 1. -/]l8of]_ | | | 1 | | 2 | | RD116b |
|  | 1. -l6= le=_ | | | 1 | | 2 | | TV116c |
|  | 1. -kmf]g_ | | | 1 | | 2 | | TL116d |
|  | 1. -lk\|mh_ | | | 1 | | 2 | | RF116e |
|  | 1. -sDKo'6/_ | | | 1 | | 2 | | CM116f |
|  | 1. -leQ] 38L_ | | | 1 | | 2 | | WC116g |
|  | 1. -Uof; luh/_ | | | 1 | | 2 | | GG116h |
|  | 1. -;f]nf/ Kofg]n_ | | | 1 | | 2 | | SP116i |
| 1. 117 | s] tkfO{sf] kl/jf/sf ;b:ox?sf] tnsf dWo] sg} ;jf/L ;fwg 5< | | | 1. -5_ | | 2. -5}g_ | |  |
|  | -;fO{sn . l/S;f_ | | | 1 | | 2 | | BC117a |
|  | -df]6/ ;fO{sn_ | | | 1 | | 2 | | MC117b |
|  | -6]Dkf]_ | | | 1 | | 2 | | TM117c |
|  | -sf/ . 6\|s_ | | | 1 | | 2 | | CR117d |
|  | -uf]? Uff9f_ | | |  | |  | | CT117e |
|  | ­­-cGo_ ­_______________ | | |  | |  | | OT117f |
| 1. 118# | -tkfO{sf] 3/df vfg]kfgL sxf“af6 NofOG5<_ -a9Ldf @ j6f pQ/x?_ | 1. -kfO{ksf] kfgL_  2. -cfugdf kfO{ksf] wf/f_  3. -;fj{hlgs . l5d]sLsf] wf/f_  4. -cfk\mg} O{gf/_  5. -;fj{hlgs . l5d]sLsf] O{gf/_  6. -cfkm\g} 6\o'j]n_  7. -;fj{hlgs . l5d]sLsf] 6\o'j]n_  8. -;txsf] kfgL_  10. -d'nsf] kfgL, s'jf_  11. -glb, vf]nf, tnfpsf] kfgL_  12. -9'+u] wf/f_  9. -cGo_ ___________________ | | | | | | WAT118 |
| 1. 119 | tkfO{sf] 3/df s:tf] k|sf/sf] rkL{ 5 < | 1. -sDaf]6 . Nkm; ug]{ vfnsf] rkL{_  2 -vfN8] rkL{_  3. -;'wfl/Psf] vfN8] rkL{_  4. -rkL{ gePsf], emf8L, v'Nnf hldg_  9. -cGo__________________ | | | | | | TOI119 |
| 1. 120 | tkfO{sf] 3/df rkL{ gePsf] eP rkL{ gagfpg'sf] sf/0f s] xf]< | 1. -k};f geP/_  2. -;do geP/_  3. -rkL{ agfpg] pko{'Qm hUuf geP/_  4. -rkL{df lbzf ug{ dg gnfUg] eP/_  5. -rkL{sf] dxTj yfxf geP/_  6. -3/ geP/ jf rkL{ aGb} u/]sf]_  9. -cGo______________________ | | | | | | NTl120 |
| 1. 121 | s] tkfO{n] k|of]u ug]{ rkL{df k|z:t kfgLsf] Joj:yf 5< | | 1. -5_  2. -5}g_ | | | | | WT121 |
| 1. 123 | s] tkfO{sf] kl/jf/ ;b:ox?dWo] sf]lx ljb]zdf sfd ul//xg'ePsf] 5<­ | | 1. -5_  2. -5}g_ | | | | | ABR123 |
| 1. 124# | olb xf] eg] s'g b]zdf sfd ul/ /xg' ePsf] 5, b]zsf] gfd n]Vg'xf];\< olb b"O{hgf ePdf b'j} n]Vg'xf];\ . | | 1 ........................................  2......................................... | | | | | CNT122a  CNT122b |
| 1. 125 | s] jxfn] lgoldt ?kdf 3/df k};f k7fpg] ug{' ePsf] 5 < | | 1. -5_  2. -5}g_  88. -ev{/} dfq uPsf], k};f k7fpg afsL_  99. -yfxf gePsf]_ | | | | | NRS125 |
| 1. 126 | -tkfO{sf] 3/df vfgf ksfpg s'g OGwg k|of]u ug'{x'G5<­_ -a9Ldf @ j6f pQ/x?_ | | | | -xf]_ | | -xf]O{g_ |  |
|  | 1. -ljB't_ | | | | 1 | | 2 | FL126a |
|  | 1. (Gas) -Uof“;_ | | | | 1 | | 2 | FL126b |
|  | 1. -uf]a/ Uof“; . afof] Uof“;_ | | | | 1 | | 2 | FL126c |
|  | 1. -dl6\6t]n_ | | | | 1 | | 2 | FL126d |
|  | 1. -bfp/f_ | | | | 1 | | 2 | FL126e |
|  | 1. -u'O7f_ | | | | 1 | | 2 | FL126f |
|  | 1. (specify) -cGo_ __________________ | | | | 1 | | 2 | FL126g |

**uef{j:yf / :ofxf/**

-o; v08sf k|Zgx? ;aeGbf kl5Nnf] lhljt aRrf ue{df ePsfaf/] ;DalGwt 5 . olb cfdf k'gM ue{jtL eO{ ;s]sLl5g\eg] ;aeGbf kl5Nnf] k6s hlGd;s]sf] lzz'af/] ;DalGwt 5 ._

| Q. # | Question | | Codes | | | | | | | |  |
| --- | --- | --- | --- | --- | --- | --- | --- | --- | --- | --- | --- |
|  | s] kl5Nnf] k6s ue{jtL x'bf s;}l;t hFfr u/fpg'eof]< | | 1. -u/fP_  2. -u/fO{g_ | | | | | | | | ANC403 |
|  | sf] sf]l;t hFfr u/fpg'eof]< **;a} hjfkmx?df uf]nf] lrGx nufpg'xf];\** . | | 1. -8fS6/_  2. -g;{ . c g dL . df= l;=sf=_  3. -x]Ny cl;:6]G6 . c x] j=_  4. -d :jf= :j= ;]=_  5. -;'8]gL_  9. -cGo_ ________________ | | | | | | | | ANC404 |
| 1. 405 | kl5Nnf] k6s ue{jtL xF'bf :jf:YosdL{af6 hDdf slt k6s hFfr ug'{eof]< | | | # -hDdf k6s_: …….  99. -yfxf 5}g_ | | | | | | | ANC405 |
| 1. 406 | kl5Nnf] k6s ue{jtL x'bf s'g s'g dlxgfdf hFfr u/fpg'eof]< | -dlxgf_ or -xKtf_ | | | | | | | Don’t remember  -yfxf 5}g_ | |  |
|  | 1. -klxnf] k6ssf] ue{jtL hFfr_ | ……… -dlxgf_ ……… -xKtf_ | | | | | | | 99 | | ANC406a |
|  | 1. -bf]>f] k6ssf] ue{jtL hFfr_ | ……… -dlxgf_ ……… -xKtf_ | | | | | | | 99 | | ANC406b |
|  | 1. -t]>f] k6ssf] ue{jtL hFfr_ | ……… -dlxgf_ ……… -xKtf_ | | | | | | | 99 | | ANC406c |
|  | 1. -rf}yf] k6ssf] ue{jtL hFfr_ | ……… -dlxgf_ ……… -xKtf_ | | | | | | | 99 | | ANC406d |
|  | 1. -kfrf} k6ssf] ue{jtL hFfr_ | ……… -dlxgf_ ……… -xKtf_ | | | | | | | 99 | | ANC406e |
|  | 1. -5}6f} k6ssf] ue{jtL hFfr_ | ……… -dlxgf_ ……… -xKtf_ | | | | | | | 99 | | ANC406f |
|  | 1. -;ftf} k6ssf] ue{jtL hFfr_ | ……… -dlxgf_ ……… -xKtf_ | | | | | | | 99 | | ANC406g |
|  | 1. -cf7f} k6ssf] ue{jtL hFfr_ | ……… -dlxgf_ ……… -xKtf_ | | | | | | | 99 | | ANC406h |
|  | 1. -gjf} k6ssf] ue{jtL hFfr_ | ……… -dlxgf_ ……… -xKtf_ | | | | | | | 99 | | ANC406i |
|  | 1. -b;f} k6ssf] ue{jtL hFfr_ | ……… -dlxgf_ ……… -xKtf_ | | | | | | | 99 | | ANC406j |
| 1. 407 | tflnsf cg';f/ slDtdf rf/ k6s ue{jtL hFfr u/]sf] xf]< lrGx nufpg'xf];\ | | | 1. -5_  2. -5}g_  77. ­-/]s8{ gePsf] _ | | | | | | |  |
| 1. 408 | s] tkfO{n] kl5Nnf] k6s ue{jtL x'bf ue{jtL hFfr sf8{ k|fKt ug{'ePsf] lyof]< | | | 1. -lyof]_  2. -lyPg_  99. -yfxf 5}g_ | | | | | | | ANC408 |
| 1. 409 | olb sf8{ k|fKt ug{'ePsf] eP xfdLnfO{ b]vfpg ;Sg'x'G5<_ | | | 1. -xf], sf8{ klg 5_  2. -xf], t/ sf8{ 5}g_  3. -xf]O{g_ | | | | | | | ANC409 |
| 1. 411# | s] tkfO{ kl5Nnf] k6s ue{jtL x'bf lgDg s'/fx? ul/Psf] lyof]< | | | | -5_ | | -5}g_ | | | -yfxf 5}g_ |  |
|  | 1. k]6 hFfr ul/Psf] lyof]< | | | | 1 | | 2 | | | 99 | ANC411a |
|  | 1. s] tkfO{n] cfO{/g rSsL kfpg'ePsf] lyof]< | | | | 1 | | 2 | | | 99 | ANC411b |
|  | 1. s] tkfO{n] h'sfsf] cf}iflw kfpg'ePsf] lyof]< | | | | 1 | | 2 | | | 99 | ANC411c |
|  | 1. s] tkfO{sf] tf}n hFfr ul/Psf] lyof]< | | | | 1 | | 2 | | | 99 | ANC411d |
|  | 1. s] tkfO{sf] prfO{ hFfr ul/Psf] lyof]< | | | | 1 | | 2 | | | 99 | ANC411e |
|  | 1. s] tkfO{sf] /Qmrfk hFfr ul/Psf] lyof]< | | | | 1 | | 2 | | | 99 | ANC411f |
|  | 1. s] tkfO{sf] lk;fa hFfr ul/Psf] lyof]< | | | | 1 | | 2 | | | 99 | ANC411g |
|  | 1. s] tkfO{sf] /ut hFfr ul/Psf] lyof]< | | | | 1 | | 2 | | | 99 | ANC411h |
| 1. 414 | s] tkfO{n] kl5Nnf] k6s ue{jtL x'bf l6= l6= vf]k lng'ePsf] lyof]< | | 1. -lyof]_  2. -lyPg_  99. -yfxf 5}g_ | | | | | | | | ANC414 |
| 1. 415 | tkfO{n] kl5Nnf] k6s ue{jtL x'bf sltj6f l6= l6= vf]k lng'ePsf] lyof]< | | -vf]k ;+Vof_ □□  77. -sf8{ gePsf]_  99. -yfxf 5}g_ | | | | | | | | ANC415 |
| 1. 416 | s] tkfO{n] kl5Nnf] k6s ue{jtL x'bf cfO{/g rSsL lbO{Psf] jf lsg]/ vfg elgPsf] lyof]< cfO{/g rSsL b]vfpg'xf];\ . | | 1. -lyof]_  2. -lyPg_  99. yfxf 5}g_ | | | | | | | | ANC416 |
| 1. 419 | s] tkfO{n] kl5Nnf] k6s ue{jtL x'bf h'sfsf] cf}iflw vfg'eof]< | | 1. -vfP_  2. -vfO{g_  99. -yfxf 5}g_ | | | | | | | | ANC419 |
| 1. 424 | tkfO{nfO{ ue{jtL hfFr ug]{af/] s;n] lg0fo  u-of]]< | | 1. -d cfkm}_  2. ->Ldfg_  3. -zf;'_  4. (संगै)  9. -cGo__________________ | | | | | | | | ANC424 |
| 1. 425 | ue{jtL cj:yfdf slt k6s :jf:YosdL{af6 hfFr u/fpg'k5{ < | | # ___________ k6s  99. yfxf 5}g | | | | | | | | ANC425 |
| 1. 426# | ue{jtL xF'bf s] s:tf nIf0fx? b]lvPdf t'?Gt :jf:YosdL{sf] ;xof]u lng'k5{ < | | 1. -cFfvf lt/ld/fP/ wldnf] b]Vg]_  2. -6fpsf] c;fWo} b'Vg]_  3. -sDk 5'6\g], a]xf]; x'g] jf d'5f{ kg]{_  4. -xft, zl// jf cg'xf/ ;'lGgg]_  5. -of]gLaf6 clnslt klg /ut cfpg] | | | | | | | | ANC426a  ANC426b  ANC426c  ANC426d  ANC426e |
| 1. 427# | kl5Nnf] k6s ue{jtL x'bf s] tkfO{nfO{ tnsfdWo] s'g} ;dZofx? eof]< | | | | | -eof]_ | | -ePg_ | | -yfxf 5}g_ |  |
|  | 1. cfvf lt/ld/fP/ wldnf] b]Vg] | | | | | 1 | | 2 | | 99 | PRB427a |
|  | 1. s8fl;t tNnf] k]6 b'Vg | | | | | 1 | | 2 | | 99 | PRB427b |
|  | 1. 6fpsf] c;fWo} b'Vg] | | | | | 1 | | 2 | | 99 | PRB427c |
|  | 1. sDk_ 5'6\g], a]xf]; x'g] jf d'5f{ kg]{ | | | | | 1 | | 2 | | 99 | PRB427d |
|  | 1. xft, zl// jf cg'xf/ ;'lGgg] | | | | | 1 | | 2 | | 99 | PRB427e |
|  | 1. of]gLaf6 clnslt klg /ut cfpg | | | | | 1 | | 2 | | 99 | PRB427f |
| 1. 428# | tL ;d:ofdf s;nfO{ hrfpg' eof]< **;a} hjfkmdf uf]nf] nufpg'xf];\ .** | | | | | -xf]_ | | -xf]O{g_ | | -yfxf 5}g_ |  |
|  | 1. 3/d} 3/]n' pkrf/ u/]sf] | | | | | 1 | | 2 | | 99 | SOL428a |
|  | 1. 3/d} cf}iflw NofP/ vfPsf | | | | | 1 | | 2 | | 99 | SOL428b |
|  | 1. c:ktfn uPsf | | | | | 1 | | 2 | | 99 | SOL428c |
|  | 1. k\|f= jf= s]= . x]= kf]= jf ;a x]= kf]= uPsf] | | | | | 1 | | 2 | | 99 | SOL428d |
|  | 1. k\|fO{e]6 lSnlgs jf gl;{ª xf]ddf uPsf] | | | | | 1 | | 2 | | 99 | SOL428e |
|  | 1. cf}iflw k;naf6 cf}iflw lsg]/ vfPsf] | | | | | 1 | | 2 | | 99 | SOL428f |
|  | 1. d= :jf= :j= ;]=l;t ;Nnfx u/]sf | | | | | 1 | | 2 | | 99 | SOL428g |
|  | 1. wfdL . emfqmLl;t ;Nnfx u/]sf | | | | | 1 | | 2 | | 99 | SOL428k |
|  | 1. gft]bf/, l5d]sL jf ;fyLl;t ;Nnfx u/]sf] | | | | | 1 | | 2 | | 99 | SOL428l |
|  | 1. s]lx klg gu/]sf] | | | | | 1 | | 2 | | 99 | SOL428m |
|  | 1. cGo -v'nfpg]_ : ______________________ | | | | | 1 | | 2 | | 99 | SOL428n |
| 1. 429 | ue{jtL hfr ug]{af/] s;af6 yfxf kfpg'eof]< | | | | | -xf]_ | | -xf]O{g_ | | -yfxf 5}g_ |  |
|  | 1. kl/jf/sf ;b:ox?af6 | | | | | 1 | | 2 | | 99 | INF429a |
|  | 1. /]l8of] l6= eL= af6 | | | | | 1 | | 2 | | 99 | INF429b |
|  | 1. :jf:YosdL{x?af6 | | | | | 1 | | 2 | | 99 | INF429c |
|  | 1. ;fyL . gft]bf/ . l5d]sL . ;d'bfoaf6 | | | | | 1 | | 2 | | 99 | INF429d |
|  | 1. ljBfno . sn]h . lzIfsx?af6 | | | | | 1 | | 2 | | 99 | INF429e |
|  | 1. d= :jf= :j= ;]=af6 | | | | | 1 | | 2 | | 99 | INF429f |
|  | 1. cGo________________________________________________ | | | | | 1 | | 2 | | 99 | INF429g |

**k|;'tL :ofxf/**

**-ca d tkfO{nfO{ k|;'tL cj:yfdf ul/g] :ofxf/af/] s]lx ;f]Wg rfxG5' ._**

| Q. # | Question | | | Codes | | | |  |
| --- | --- | --- | --- | --- | --- | --- | --- | --- |
| 1. 501 | tkfO{sf] ;aeGbf kl5Nnf] aRrf sxf hGd]sf]] xf]< | | | 1. -c:ktfn_  2. -k|f= :jf= s]=_  3. -x]= kf]=+_  4. -k|fO{e]6 lSnlgs, gl;{ª xf]d_  5. -3/_  6. jf:Yo ;+:yf n}hfbf af6}df_  9. (specify) -cGo_ ______________ | | | | DEL501 |
| 1. 502# | tkfO{sf] kl5Nnf] aRrf hGdg] a]nfdf s;n] ;xof]u u/]sf] lyof]<  **;a} hjfkmdf uf]nf] nufpg'xf];\ .** _ | | | 1. -8fS6/_  2. -g;{ . c g dL . df= l;=sf=_  3. -x]Ny cl;:6]G6 . c x] j_  4. -d :jf= :j= ;]=_  5. -;'8]gL_  6. -;fyL, l5d]sL_  7. -zf;'_  8. -PSn}_  10. -dfO{tLlt/sf ;b:ox?, cfdf, lbbL_  9. cGo ________________  99. -yfxf 5}g_ | | | | DEL502 |
| 1. 504 | s] tkfO{sf] kl5Nnf] aRrf ck|];g u/]/ lgsflnPsf] xf]< | | | | 1. -xf]_  2. -xf]O{g_ | | | DEL504 |
| 1. 506 | tkfO{sf] kl5Nnf] aRrf hGdg] a]nfdf sxFf aRrf hGdfpg] eg]/ s;n] lg0f{o u/]sf] lyof]< | | 1. -cfkm}_  2. -zf;'_  3. -z;'/f_  4. ->Ldfg_  5. -cfdf . a'af_  6. -cGo gft]bf/_  9. cGo ________________  99. -yfxf 5}g_ | | | | | DEC506 |
| 1. 507# | aRrf hGdg] a]nfdf s] s:tf nIf0fx? b]lvPdf t'?Gt} :jf:Yo ;+:yfdf n}hfg'k5{< **;a} hjfkmdf uf]nf] nufpg'xf];\ .** | | 1. -* 306feGbf nfdf] ;'Ts]/L Joyf nfUg]_  2. -lzz' hGdg'eGbf klxn] xft lg:sg]_  3. -lzz' hGdg'eGbf klxn] v'§f lg:sg]_  4. -lzz' hGdg'eGbf klxn] gfn lg:sg]]_  5. -lzz' hGdg'eGbf klxn] jf kl5 cToflws /Qm>fj x'g]_  6. -aRrf hGdfpg] a]nfdf sDk 5'6\g]_  9. cGo ________________  99. -yfxf 5}g_ | | | | | INF507a  INF507b  INF507c  INF507d  INF507e  INF507f  INF507g  INF507h |
| 1. 508 | tkfO{sf] kl5Nnf] aRrf hGdfpg] a]nfdf tnsfdWo] s'g} nIf0fx? ePsf] lyof]< | | | | | -5_ | -5}g_ |  |
|  | 1. -w]/} /Qm>fj ePsf]_ | | | | | 1 | 2 | PRB508a |
|  | 1. -w]/} Hj/f] cfPsf]_ | | | | | 1 | 2 | PRB508a |
|  | 1. -kfgL kmf]sf km'6]sf] @$ 306f;Dd klg aRrf ghlGdPsf]_ | | | | | 1 | 2 | PRB508b |
|  | 1. -sDk 5'6]sf]_ | | | | | 1 | 2 | PRB508c |
|  | 1. -;fx\|}l;t 6fpsf] b'v]sf]_ | | | | |  |  | PRB508d |
|  | 1. -* 306f eGbf al9;Dd Joyf nfu]sf]_ | | | | | 1 | 2 | PRB508e |
|  | 1. -xft, gfn jf v'§f klxn] lg:ssf]_ | | | | | 1 | 2 | PRB508f |
|  | 1. -;fn gem/]sf]_ | | | | | 1 | 2 | PRB508g |
|  | Tof] ;d:ofdf tkfO{n] s] ug{'eof]< **;a} hjfkmdf uf]nf] nufpg'xf];\ .** _ | 1. -3/]n' pkrf/_  2. -3/d} cf}iflw NofP/ vfP_  3. -c:ktfn_  4. -k|f= :jf= s]= . x]= kf]= . ;a x]= kf]=_  5. -k|fO{e]6 lSnlgs, gl;ª xf]d_  6. -d= :jf= :j= ;]=l;t k/fdz{ u/]+_  7. -:jf:YosdL{l;t k/fdz{ u/]+_  8. -wfdL . emfqmL nufP_  10. -gft]bf/, l5d]sL, ;fyLl;t k/fdz{ u/]+_  11. -s]lx klg ul/g_  9. -cGo_ ________________ | | | | | | SOL509 |

# Multiple answers possible

**gjhft lzz'sf] :ofxf/**

**ca d tkfO{sf] aRrfnfO{ ! dlxgfleq u/]sf] :ofxf/af/] ;f]Wg rfxG5' .**

| Q. # | Question | | Codes |  |
| --- | --- | --- | --- | --- |
|  | aRrf lhljt hGd]sf] lglZrt ug'{xf];\ .lhjLt aRrf hGd]sf | | 1. -xf]]_  2. -xf]O{g_  99. -yfxf 5}g_ | LIV701 |
|  | s] lzz' hGdg] a]nfdf ;'Ts/L ;fdu|Lsf] a6\6f k|of]u ul/Psf] lyof]<_ | | 1. -xf]]_  2. -xf]O{g_  99. -yfxf 5}g_ | CDK702 |
|  | lzz'sf] gfn s] n] sfl6Psf] lyof]< | | 1. -gof kQL_  2. -k'/fgf] kQL t/ pdfn]/_  3. -k'/fgf] kQL gpdfnLsg_  4. -rSs'_  5. -xl;of_  6. -v's'/L_  7. -s}rL_  8. -c:ktfndf hlGdPsf] / s] u-of] yfxf gePsf]_  9. -cGo_________________  99. -yfxf 5}g_ | NNC703 |
| 1. 704 | s] lzz' hGdg] lalQs} t/ ;fn gemb}{ gfn sfl6Psf] xf]< | | 1. -xf]]_  2. -xf]O{g_  99. -yfxf 5}g_ | NNC704 |
| 1. 705 | lzz'sf] gfeLdf s] n] aflwPsf] lyof]< | | 1. -gof wfuf]_  2. -pdfn]sf] wfuf]_  3. -gpdfn]sf] wfuf]_  4. -lSnk_  9. -cGo_________________  99. -yfxf 5}g_ | NNC705 |
| 1. 706 | s] lzz'sf] gfn sfl6;s]kl5 gfeLdf s]lx nufO{Psf] lyof] < | | 1 -xf]]_  2. -xf]O{g_  99. -yfxf 5}g_ | NNC706 |
| 1. 707# | gfn sfl6;s]kl5 s] nufO{Psf] lyof]< cGo s]lx nufO{Psf] lyof]< **;a} hjfkmdf uf]nf] nufpg'xf];\ .** | | 1. -Snf]x]{Shfl8g gfeL dNxd_  2. -t]n_  3. -v/fgL_  4. -l;Gb'/_  5. -dNxd . kfp8/_  6. -uf]a/_  7. -a];f/_  8. -l3p_  9. -cGo__________________  99. -yfxf 5}g_ | NNC707 |
| 1. 708 | s] tkfO{sf] aRrfnfO{ hGdg] lalQs} t/ ;fn gemb}{ Gofgf] sk8fn] a]/]/ /flvPsf] lyof]< | | 1. -xf]]_  2. -xf]O{g_  99. -yfxf 5}g_ | NNC708 |
| 1. 709 | aRrf hGd]sf] slt ;dokl5 lzz'nfO{ g'xfO{Psf] lyof] < | | 1. -! 306fleqdf_  2. -@ b]lv @$ 306fleqdf_  3. -@$ 306fkl5_  99. -yfxf 5}g_ | NNC709 |
| 1. 710 | s] tkfO{n] kl5Nnf] aRrfnfO{ slta]nf ;aeGbf klxnf] k6s cfkm\gf] b'w v'jfpg' ePsf] lyof]< | | 1. -t'?Gt}, aRrf hGd]sf] ! 306fleqdf_  2. -aRrf hGd]sf] ! 306fkl5_  9. -cGo__________________  99. -yfxf 5}g_ | NNC710 |
| 1. 711 | s] tkfO{n] kl5Nnf] aRrfnfO{ lauf}tL b'w -klxnf] b'w_ v'jfpg' ePsf] lyof]< | | 1. -xf]]_  2. -xf]O{g_  99. -yfxf 5}g_ | NNC711 |
| 1. 712 | s] tkfO{ aRrfnfO{ cem} b'w r';fO{ /xg'ePsf] 5< | | 1. -xf]]_  2. -xf]O{g_ | NNC712 |
| 1. 713 | lzz' hGd]sf] slt ;dokl5 g'xfO{lbg'k5{< | 1. -aRrfhGd]kl5 t'?Gt}_  2. -aRrf hGd]sf] @$ 306fleqdf_  3. -aRrf hGd]sf] @$ 306fkl5 dfq_  4. -g'xfO{lbg' x'b}g_  9. -cGo__________________  99. -yfxf 5}g_ | | NNC713 |
| 1. 714 | lzz' hGd]sf] slt ;dokl5 cfdfs]f b'w v'jfpg'k5{< | 1. -aRrfhGd]kl5 t'?Gt}_  2. -;fn em/]kl5_  3. -aRrfnfO{ g'xfO{;s]kl5_  4. aRrf hGd]sf] @$ 306fkl5_  9. -cGo_ _________________  99. -yfxf 5}g_ | | NNC714 |

| 1. 720 | gjhft lzz'df b]vfkg{;Sg] vt/fsf nIf0fx? s] s] x'g< | -xf]_ | -xf]Og_ | -yfxf 5}g_ |  |  |
| --- | --- | --- | --- | --- | --- | --- |
|  | 1. -Hj/f] cfpg]_ | 1 | 2 | 99 | DS720a |  |
|  | 1. -cfdfsf] b'w gr':g] _ | 1 | 2 | 99 | DS720b |  |
|  | 1. -l56f] l56f] zf; km]g]{_ | 1 | 2 | 99 |  |  |
|  | 1. -s8f sf]vf xfGg]_ | 1 | 2 | 99 | DS720d |  |
|  | 1. -;':t, a]xf]; jf sd rnfO{_ | 1 | 2 | 99 | DS720e |  |
|  | 1. -5fnfdf lkk el/Psf kmf]sfx? b]lvg]_ | 1 | 2 | 99 | DS720f |  |
|  | 1. -gfO{6f] kfSg]_ | 1 | 2 | 99 | DS720g |  |
|  | 1. -lr;f] jf l;tf+u x'g]_ | 1 | 2 | 99 | DS720h |  |
| 1. 721 | s] tkfO{sf] lzz'nfO{ hGd]sf] ! dlxgfleqdf lgDgdWo] s'g} ;dZof eof]< **;a} hjfkmdf uf]nf] nufpg'xf];\ .** | -eof]_ | -ePg_ | -yfxf 5}g_ |  |  |
|  | 1. -Hj/f] cfpg]_ | 1 | 2 | 99 | PRB721a |  |
|  | 1. -:tgkgfsf] ;dZof_ | 1 | 2 | 99 | PRB721b |  |
|  | 1. -l56f] l56f] zf; km]g]{_ | 1 | 2 | 99 | PRB721c |  |
|  | 1. -sf]vf xfGg]_ | 1 | 2 | 99 | PRB721d |  |
|  | 1. -;':t jf a]xf];_ | 1 | 2 | 99 | PRB721e |  |
|  | 1. -5fnfdf lkk el/Psf] kmf]sf b]lvg]_ | 1 | 2 | 99 | PRB721f |  |
|  | 1. -gfeLsf] jl/k/L /ftf] x'g] jf gfeL kfSg]_ | 1 | 2 | 99 | PRB721g |  |
|  | 1. -lr;f] jf l;tf+u x'g]_ | 1 | 2 | 99 | PRB721h |  |
| 1. 722# | oL ;dZofdf tkfO{n] s] ug{'eof] jf s;sf] ;xof]u lng'eof]< **;a} hjfkmdf uf]nf] nufpg'xf];\ .** | -xf]_ | -xf]Og_ | -yfxf 5}g_ |  |  |
|  | 1. -3/]n' pkrf/ u/]+_ | 1 | 2 | 99 | SOL722a |  |
|  | 1. -3/df cf}iflw v'jfP_ | 1 | 2 | 99 | SOL722b |  |
|  | 1. -c:ktfn nu]+ jf 8fS6/nfO{ hrfP_ | 1 | 2 | 99 | SOL723c |  |
|  | 1. -k\|f= :jf= s]=, x]= kf]= df hrfP_ | 1 | 2 | 99 | SOL723d |  |
|  | 1. -lghL lSnlgs . gl;{ª xf]ddf hrfP_ | 1 | 2 | 99 | SOL723e |  |
|  | 1. -cf}iflw k;naf6 cf}iflw lsg]/ v'jfP_ | 1 | 2 | 99 | SOL723f |  |
|  | 1. -d= :jf= :j= ;]=nfO{ b]vFfP_ | 1 | 2 | 99 | SOL723g |  |
|  | 1. -wfdL . emfqmL nuFfP_ | 1 | 2 | 99 | SOL723h |  |
|  | 1. -gft]bf/ . l5d]sLnfO{ b]vfP_ | 1 | 2 | 99 | SOL723i |  |
|  | 1. -s]lx klg ul/g_ | 1 | 2 | 99 | SOL723j |  |
|  | 1. (specify) -cGo_: _____________________ | 1 | 2 | 99 | SOL723k |  |
